# Expression of a stress-inducible heme oxygenase-1 in NK cells is maintained in the process of human aging

**DOI:** 10.3389/fimmu.2024.1398468

**Published:** 2024-07-19

**Authors:** Lucyna Kaszubowska, Jan Jacek Kaczor, Mateusz Jakub Karnia, Jerzy Foerster, Zbigniew Kmieć

**Affiliations:** ^1^ Department of Histology, Medical University of Gdańsk, Gdańsk, Poland; ^2^ Department of Animal and Human Physiology, University of Gdańsk, Gdańsk, Poland; ^3^ Department of Social and Clinical Gerontology, Medical University of Gdańsk, Gdańsk, Poland

**Keywords:** heme oxygenase-1, NK cells, CD3-CD56+ cells, HSP32, interleukin 6, NLRP3, oxidative stress, aging

## Abstract

**Introduction:**

Heme oxygenase-1 (HO-1) is a stress-inducible heat shock protein (HSP32) that exerts cytoprotective effects against oxidative stress and inflammation, and is involved in the maintenance of cellular homeostasis. This study aimed to evaluate the expression of HO-1 in natural killer (NK) cells from individuals of different age groups after stimulation with various factors, and to analyze the relationships between the concentration of this cytoprotective protein and parameters corresponding to oxidative stress and inflammation, that is, NOD-like receptor protein 3 (NLRP3), glutathione (GSH), GSH disulfide (GSSG), and interleukin 6 (IL-6).

**Methods:**

The study population comprised three age groups: young adults (age range, 19–23 years), older adults aged under 85 years (age range, 73–84 years), and older adults aged over 85 years (age range, 85–92 years). NLRP3, GSH, and GSSG concentrations were measured in serum, whereas the HO-1 concentration and IL-6 expression were studied in NK cells cultivated for 48 h and stimulated with IL-2, lipopolysaccharide (LPS), or phorbol 12-myristate 13-acetate (PMA) with ionomycin.

**Results:**

The analysis of serum NLRP3, GSH, and GSSG concentrations revealed no statistically significant differences among the studied age groups. However, some typical trends of aging were observed, such as a decrease in GSH concentration and an increase in both GSSG level, and GSSG/GSH ratio. The highest basal expression of IL-6 and lowest basal content of HO-1 were found in NK cells of adults over 85 years of age. The NK cells in this age group also showed the highest sensitivity to stimulation with the applied factors. Moreover, statistically significant negative correlations were observed between HO-1 and IL-6 expression levels in the studied NK cells.

**Conclusions:**

These results showed that NK cells can express HO-1 at a basal level, which was significantly increased in activated cells, even in the oldest group of adults. The reciprocal relationship between HO-1 and IL-6 expression suggests a negative feedback loop between these parameters.

## 1 Introduction

NK cells are large granular lymphocytes belonging to the family of group 1 innate lymphocytes (ILC1). They play a pivotal role in the first line of defense against infections and developing malignancies, as well as in the immune surveillance of senescent cells, which accumulate with age and likely contribute to chronic low-grade inflammation that develops with aging (1). Aging is a multifactorial process that affects all cells and organs in the human body, resulting in progressive functional impairment and loss of homeostasis. One of the most dysregulated systems during the aging process is the immune system, with alterations occurring in both innate and adaptive immunity (2). NK cells, similar to adaptive and other innate immune cells, undergo a series of changes that comprise the process of immunosenescence, which manifests as age-related cell dysfunction that, in NK cells, is characterized by reduced cytokine secretion and decreased target cell cytotoxicity accompanied by an increased cell number. The latter attribute is hypothesized to compensate for age-related loss in the effector function of NK lymphocytes at the individual cell level (3, 4). The preservation of NK cell features until advanced age has previously been proposed to contribute to longevity and successful aging (4–6).

During aging, the imbalance between cellular oxidants and antioxidants increases exponentially. Oxidative stress increases because antioxidant defense mechanisms do not counterbalance age-related increase in reactive oxygen species (ROS) generation. The balance between ROS and antioxidants shifts progressively towards a more pro-oxidant state. The oxidant/antioxidant system is quantitatively dependent on the ratios of interconvertible (reduced and oxidized) redox couples, such as glutathione (GSH) and GSH disulfide (GSSG), NADH/NADPH, and cysteine/cystine, among others. However, the GSH/GSSG couple is regarded as the principal intracellular determinant of the redox state because it is more abundant than other redox couples. The redox potential of this couple depends on the amount of GSH and GSSG, and the ratio between these parameters (7).

Cumulative oxidative stress and chronic inflammation have been found to play essential roles in age progression (8, 9). Alterations in the redox status, accumulation of damaged molecules driven by oxidative stress, and dysregulation of the immune system ultimately lead to inflammaging. This low-grade, chronic inflammatory state usually results from the impaired elimination of damaged cells or macromolecules and pro-inflammatory cytokines secreted by senescent cells (10). Inflammaging is also characterized by impairment of the balance between stimulatory and regulatory mediators, such as cytokines or acute-phase reactants (8, 11, 12). The age-related state of chronic inflammation may be counterbalanced to a certain extent by anti-inflammatory protective mechanisms. However, when efficient control is lost, the inflammatory process increases, leading to tissue damage and the development of age-associated diseases (11, 13).

The NOD-like receptor protein 3 (NLRP3) inflammasome is a protein complex that plays a crucial role in controlling innate immune responses. It has the broadest functional scope of all inflammasomes and can respond to a wide range of agonists that arise during aging. The NLRP3 inflammasome can be activated via canonical, non-canonical, and alternative pathways (14). Factors acting as priming signals for the NLRP3 inflammasome include danger-associated molecular patterns (DAMPs), such as ATP, uric acid, oxidatively modified DNA, and aggregated proteins that occur as a result of cellular turnover or cell stress (15). Pathogen-associated molecular patterns (PAMPs) are the other source of priming signals for NLRP3 inflammasome in the canonical pathway. Non-canonical activation of the NLRP3 inflammasome is mainly mediated by lipopolysaccharide (LPS) that enters the cytoplasm via endocytosis without the involvement of toll-like receptor 4 (TLR4) (16). Intracellular LPS directly binds to the caspase 4 CARD domain in human cells, promoting K^+^ efflux, and activating the NLRP3 inflammasome. In contrast to the canonical and non-canonical pathways, the alternative pathway is not dependent on K^+^ efflux, and IL-1 is released gradually, as opposed to the all-or-nothing response during canonical activation (14). Priming and subsequent activation are the two steps in the canonical activation pathway. The priming step is crucial for expression of the *NLRP3* gene, the product of which is the sensor protein of the NLRP3 inflammasome complex, comprising also an adaptor protein, an apoptosis-associated speck-like protein containing a caspase recruitment domain (ASC), and the effector enzyme, caspase 1 (17). Inflammaging primes the NLRP3 inflammasome signaling cascade via various mechanisms (18).

Priming signals also activate cellular stress response pathways involving heme oxygenase-1 (HO-1), which was shown to inhibit inflammasome activation (19). Heme oxygenases responsible for the degradation of heme groups into carbon monoxide, free ferrous iron, and biliverdin, are found in mammals in two different isoforms (HO-1 and HO-2) (20). The HO-3 isoform has also been described in rat; however, it was found to be encoded by pseudogenes derived from the HO-2 gene, and no functional HO-3 protein has been discovered (21). HO-2 is constitutively expressed and found mainly in the brain and testis, whereas HO-1 is a stress-inducible protein that shows a predominantly low level of expression when cells are at the resting stage; however, its expression increases dramatically in the presence of heavy metals, heme, cytokines, hormones, lipid metabolites, and changes in oxygen status (20, 22). HO-1 is associated with the smooth endoplasmic reticulum, but is also present in the cell nucleus, mitochondria, and caveolae. This stress-responsive protein has a molecular weight of 32.8 kDa and belongs to the family of heat shock proteins (HSPs). HSP32 exerts cytoprotective effects against oxidative stress, inflammation, and other types of cellular stress (23).

Interleukin 6 (IL-6) is a pleiotropic cytokine involved not only in inflammatory responses, but also in the regulation of metabolic, regenerative, and neural processes, exhibiting both pro- and anti-inflammatory activities. It can be synthesized and secreted by many cell types, including T cells, innate immune cells (circulating dendritic cells, neutrophils, NK cells, monocytes, eosinophils, basophils, tissue-resident mast cells, and macrophages), fibroblasts, endothelial cells, and hepatocytes (24, 25). IL-6 is usually present at low levels in the blood; however, its serum concentration increases during aging and in subjects with markers of frailty and chronic disease, in whom its levels typically correspond with mortality (26). Depending on the cell type under study, a feedback circuit between IL-6 and HO-1 has previously been described; it is positive in human multiple myeloma cells (27) and negative in hepatoma cells (22).

The aim of our study was to evaluate the expression of HO-1 in non-stimulated and stimulated (with various factors) NK cells of different age groups and to analyze the relationships between the intracellular concentration of HO-1 and parameters corresponding to oxidative stress and inflammation (i.e., NLRP3, GSH, GSSG, and IL-6). HSP32 is a stress-inducible protein with both antioxidant and anti-inflammatory properties and is involved in the maintenance of cellular homeostasis, which is usually disturbed during aging (28, 29). However, this has not yet been described in NK cells, which are important components of innate immunity and healthy aging in humans (3, 30). We also examined the possibility of a relationship between HO-1 and IL-6 expression, as this crosstalk was observed in other types of immune and non-immune cells (22). This study refers to our previous research on the expression of cellular protective proteins in NK cells associated with aging, which concerned sirtuin 1 (SIRT1), HSP70, and manganese superoxide dismutase (SOD2) (31), as well as α-Klotho, another protein that has both anti-inflammatory and antioxidative properties, and is also involved in numerous signaling pathways activated during aging (32).

## 2 Materials and methods

### 2.1 Participants

Forty volunteers aged 19–92 years (33 women and 7 men) participated in this study. The exclusion criteria included CRP >5 mg/L, cancer, autoimmune disease, diabetes, infection, use of immunosuppressors, glucocorticoids or nonsteroidal anti-inflammatory drugs, and moderate-to-severe dementia, which was assessed using the “Mini Mental State Examination,” with a score below 23 points (33). The geriatric conditions of older adults were also considered by applying Katz’s scale to assess “activities of daily living”, and only individuals with 5–6 points were enrolled in this study (34). Older adults were volunteers recruited from local continuing care retirement communities, and young adults were students at the Medical University of Gdańsk, Poland. The participants were subdivided into three groups: 14 young adults aged 19–23 (mean age, 20.5 ± 0.3 years; 11 women and 3 men); 13 older adults aged 73–84, referred to in this study as “older adults under 85” (mean age, 78.3 ± 1.0 years; 10 women and 3 men); and 13 older adults aged 85–92, referred to in this study as “older adults over 85” (mean age, 87.2 ± 0.5 years; 12 women and 1 man). All volunteers provided informed consent, and the study was approved by the Bioethics Committee for Scientific Research of the Medical University of Gdańsk, Poland (NKEBN/225/2010). The immunological characteristics of the study population have been previously described (35).

### 2.2 Assessment of the serum concentration of NLRP3

The concentration of NLRP3 protein in serum was established using the Human NLRP3 SimpleStep ELISA Kit (Abcam, Cambridge, UK), following the manufacturer’s instructions. All samples were run in duplicate, and the NLRP3 concentration in serum was expressed as ng/mL and ng/mg serum protein, whose concentration was measured with the Pierce BCA Protein Assay Kit (Thermo Fisher Scientific, Waltham, MA, USA).

### 2.3 Determination of GSH and GSSG serum concentrations and the GSSG/GSH ratio

Serum concentrations of GSH and GSSG, as well as the GSSG/GSH ratio, were determined using a Glutathione Assay Kit (Cayman Chemical, Ann Arbor, MI, USA), according to the manufacturer’s instructions. All samples were run in duplicate. GSH and GSSG concentrations in serum were expressed in μM.

### 2.4 Preparation of peripheral blood mononuclear cell cultures

Peripheral blood mononuclear cells (PBMCs) were isolated from venous blood samples collected in tubes containing EDTA with conventional Ficoll-Uropoline density gradient centrifugation. PBMCs were then washed and resuspended in RPMI-1640 medium supplemented with 5% FBS, penicillin (100 U/mL)–streptomycin (100 μg/mL), and 2-mercaptoethanol (5 × 10^−5^ M) (all purchased from Sigma-Aldrich, St. Louis, MO, USA). The expression of intracellular IL-6 was investigated in PBMCs cultivated for 48 h *in vitro* (5 × 10^5^ cells/0.5 mL), which were stimulated with IL-2 (100 U/mL) (BD Biosciences, San Jose, CA, USA), LPS (1 μg/mL) (Sigma-Aldrich), or PMA (50 ng/mL) with ionomycin (500 ng/mL) (Sigma Aldrich) at the final 5 h of cell culture or were left without stimulation (control). Simultaneously, Golgi Stop reagent (0.5 μL/well in 0.5 mL medium; BD Biosciences) was added to PBMC cultures to stop extracellular export of the cytokine. PBMCs were then collected and washed with 1 mL BD Staining Buffer.

### 2.5 Staining of surface and intracellular antigens for flow cytometry in peripheral blood mononuclear cells

PBMCs (2.5 × 10^5^ cells) were aliquoted into flow cytometry tubes, and CD3-FITC-conjugated (0.125 μg/mL; clone UCHT1) (BD Biosciences) and CD56-APC-conjugated (0.6 μg/mL; clone NCAM16.2) (BD Biosciences) monoclonal antibodies added for cell surface antigen staining. After 30 min of incubation in the dark at room temperature (20-25°C), the cells were washed twice with 1 mL BD Staining Buffer (PBS without Ca^2+^ and Mg^2+^, 1% FBS, 0.09% sodium azide) and resuspended in 0.25 mL Fixation/Permeabilization Solution for 20 min at 4°C, following the manufacturer’s protocol (BD Cytofix/Cytoperm Fixation/Permeabilization Kit). Cells were washed twice with 1 mL BD Perm/Wash buffer and relevant volumes of IL-6-PE-conjugated (0.25 μg/mL; clone MQ2-13A5) (BD Biosciences) monoclonal antibodies were added for staining of intracellular antigens, following the manufacturer’s instructions. After 30 min of incubation in the dark at room temperature (20-25°C), the cells were washed twice with 1 mL BD Perm/Wash buffer and resuspended in Staining Buffer prior to flow cytometric analysis. Samples were run on a BD FACSCalibur flow cytometer equipped with an argon ion laser (488 nm), and data evaluated with BD CellQuest Pro software (BD Biosciences) after collecting 10,000 gated events (lymphocytes). Peripheral blood lymphocytes were gated using forward- (FCS) and side-scatter (SSC) parameters. NK cells were identified as CD3^-^CD56^+^ cells and analyzed for the frequency of cells expressing IL-6 (presented in the Results section as a percentage of NK cells expressing IL-6). Relevant isotype controls were included in the analysis.

### 2.6 Preparation of NK cell cultures

NK cells (CD3^−^CD56^+^) were isolated from PBMCs via negative selection with the Human NK Cell Enrichment Kit and EasySep Magnet (Stemcell Technologies, Vancouver, Canada), according to the manufacturer’s instructions. The purity of the enriched NK cell fractions analyzed using flow cytometry was almost 95%. NK cells (1 × 10^5^/0.1 mL) were then cultivated in RPMI-1640 medium supplemented with 5% FBS, penicillin (100 U/mL)–streptomycin (100 μg/mL), and 2-mercaptoethanol (5 × 10^−5^ M) for 48 h in the absence (control) or presence of IL-2 (100 U/mL), LPS (1 μg/mL), or PMA (50 ng/mL) with ionomycin (500 ng/mL). The cells were then centrifuged into a tightly packed pellet, the culture medium removed, and the cells snap-frozen in liquid nitrogen and stored at −80°C for further analysis.

### 2.7 Determination of HO-1 concentrations in NK cells

The concentrations of HO-1 in the cell extracts of NK cells unstimulated or stimulated for 48 h with IL-2, LPS, or PMA with ionomycin were determined using the Human Heme Oxygenase SimpleStep ELISA Kit (Abcam), according to the manufacturer’s protocol. All samples were run in duplicate, and HO-1 concentrations in the cell extract samples expressed in pg/mL.

### 2.8 Statistics

All data are expressed as the mean ± SEM. Normality of the data distribution was analyzed using the Shapiro–Wilk test. The Kruskal–Wallis test was used to compare experimental data for nonparametric distribution in the analysis of three or more independent groups. Dunn’s test was used for pairwise multiple comparisons between independent groups, and the Wilcoxon signed-rank test used to compare two related groups. The Spearman correlation coefficient (Rs) was used to determine the strength of the relationship between variables (Statistica, version 13; Statsoft, Tulsa, OK, USA). Differences or correlations were considered statistically significant at p <0.05.

## 3 Results

### 3.1 NLRP3 inflammasome protein levels in serum

**Figure 1:**
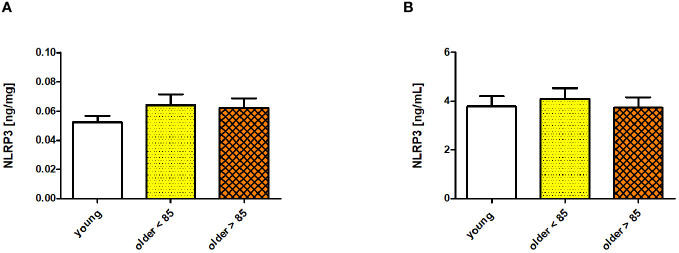
NLRP3 concentrations measured in sera of young adults, older adults aged under 85 years, and older adults aged over 85 years. Data are presented as the mean ± SEM; p >0.05 (compared using the Kruskal–Wallis test). **(A)** Concentration of NLRP3 in serum presented in ng/mg protein. **(B)** Serum NLRP3 concentration presented in ng/mL.

The serum concentration of NLRP3 was measured in all the studied age groups (young adults, older adults under 85 years, and older adults over 85 years). The NLRP3 protein levels did not differ significantly among the compared groups; however, some variability was observed. The lowest NLRP3 concentration was observed in the group of young adults (0.052 ± 0.004 ng/mg), with higher concentrations measured in both groups of older adults (0.064 ± 0.007 ng/mg in adults under 85 years and 0.062 ± 0.006 ng/mg in adults over 85 years) when expressed in ng/mg sample protein (**Figure 1A**). The NLRP3 concentrations were comparably lower in the group of young adults (3.793 ± 0.422 ng/mL) and older adults over 85 years (3.749 ± 0.424) when expressed in ng/mL. The concentration increased only in the group of older adults aged under 85 years (4.105 ± 0.444 ng/mL) (**Figure 1B**). Scatter plots of the data are shown in **Supplementary Figure 1**.

### 3.2 GSH, GSSG, and the GSSG/GSH ratio in serum

**Figure 2:**
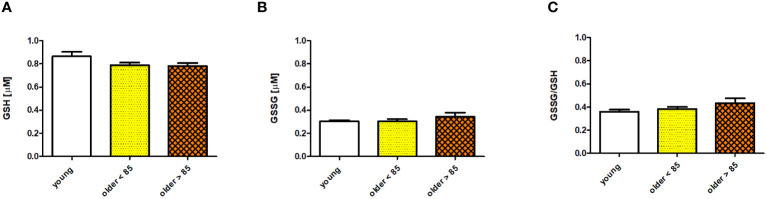
Selected parameters of the antioxidant/oxidant system determined in sera of young adults, older adults aged under 85 years, and older adults aged over 85 years. Data are presented as the mean ± SEM; p >0.05 (compared using the Kruskal–Wallis test). **(A)** Concentration of glutathione (GSH) in serum (µM). **(B)** Concentration of glutathione disulfide (GSSG) in serum (µM). **(C)** Serum GSSG/GSH ratio.

Serum concentrations of GSH and GSSG and the GSSG/GSH ratio measured in young adults and both groups of older adults showed no age-related, statistically significant differences; however, some disparities were observed. The GSH concentration was the highest in the group of young adults (0.865 ± 0.039 μM) and comparably lower in both groups of older adults (0.79 ± 0.022 and 0.782 ± 0.025 μM in adults under and over 85 years, respectively) (**Figure 2A**). In contrast, GSSG was comparably lower in the group of young adults (0.304 ± 0.009 μM) and older adults under 85 years (0.304 ± 0.019 μM), and was slightly increased in older adults over 85 years (0.342 ± 0.037 μM) (**Figure 2B**). The GSSG/GSH ratio was calculated as the lowest in the group of young adults (0.359 ± 0.017), higher in older adults under 85 years (0.383 ± 0.018), and the highest in older adults over 85 years (0.434 ± 0.04) (**Figure 2C**). Scatter plots of the data are shown in **Supplementary Figure 2**.

### 3.3 Expression of IL-6 in CD3^−^CD56^+^ cells

**Figure 3:**
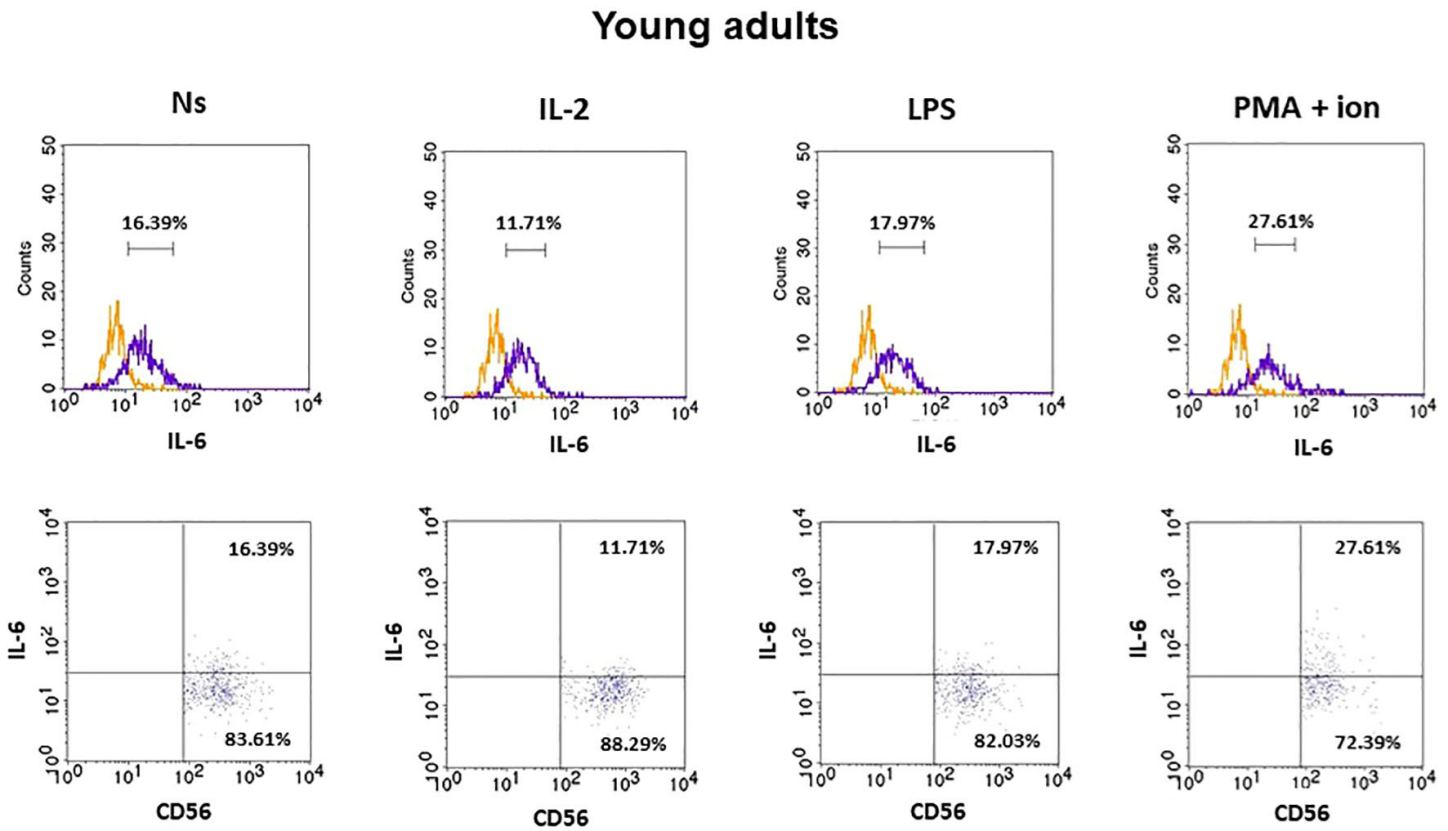
FACS histogram overlay plots (upper) for IL-6 expression in NK cells (CD3^−^CD56^+^) presented in the representative samples of young adults with corresponding dot plots (lower). Each overplay plot shows graphs depicting the isotype control (orange line) and respectively unstimulated NK cells (Ns), NK cells stimulated with IL-2 (IL-2), LPS (LPS), and PMA with ionomycin (PMA + ion) (purple line).

**Figure 4:**
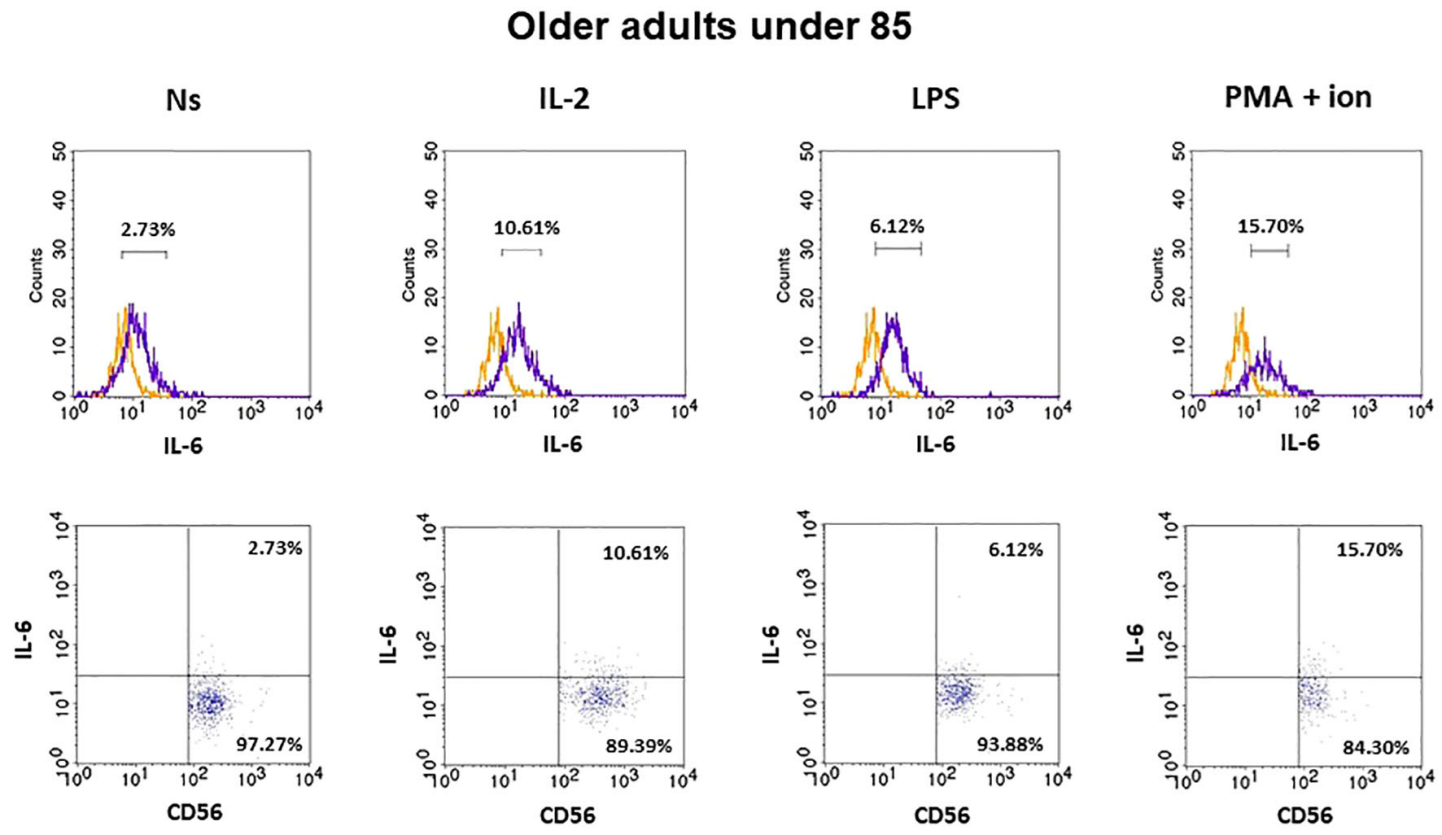
FACS histogram overlay plots (upper) for IL-6 expression in NK cells (CD3^-^CD56^+^) presented in the representative samples of older adults aged under 85 years with corresponding dot plots (lower). Each overlay plot shows graphs depicting the isotype control (orange line) and respectively unstimulated NK cells (Ns), NK cells stimulated with IL-2 (IL-2), LPS (LPS), and PMA with ionomycin (PMA + ion) (purple line).

**Figure 5:**
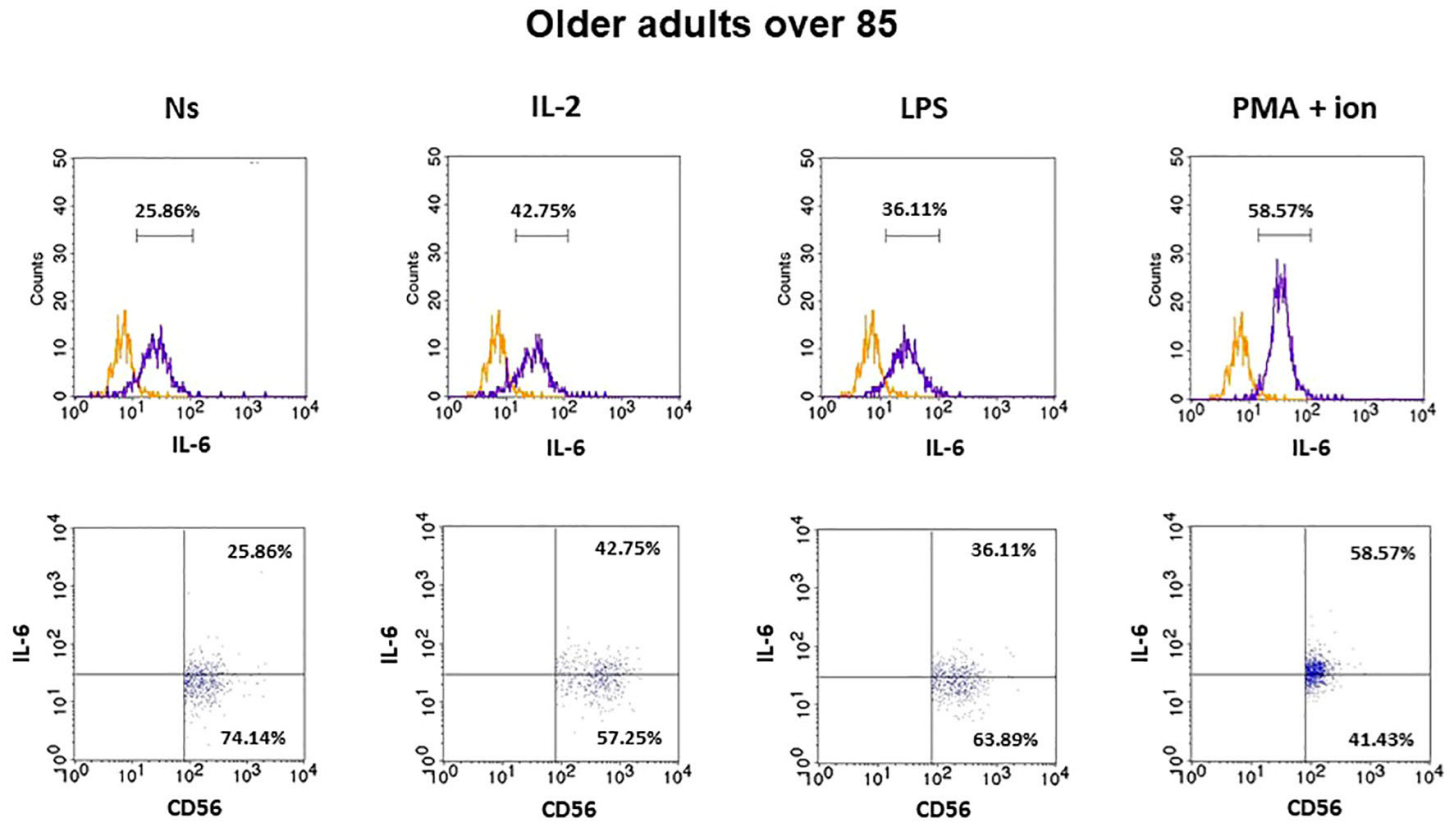
FACS histogram overlay plots (upper) for IL-6 expression in NK cells (CD3^-^CD56^+^) presented in the representative samples of older adults aged over 85 years with corresponding dot plots (lower). Each overlay plot shows graphs depicting the isotype control (orange line) and respectively unstimulated NK cells (Ns), NK cells stimulated with IL-2 (IL-2), LPS (LPS), and PMA with ionomycin (PMA + ion) (purple line).

**Figure 6:**
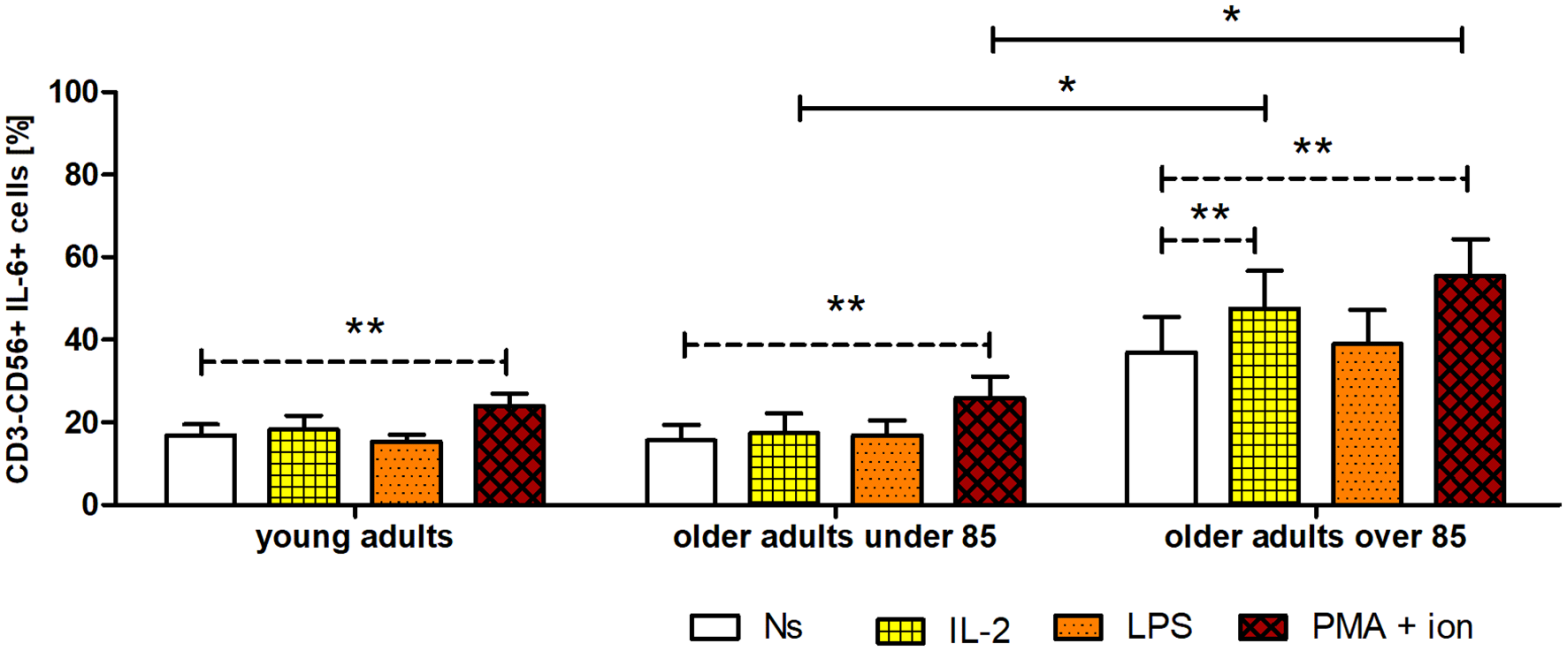
Expression of IL-6 in cultured, non-stimulated, and stimulated (IL-2, LPS, or PMA with ionomycin) NK cells of young adults, older adults aged under 85 years, and older adults aged over 85 years. Data are presented as the mean ± SEM and show the expression of IL-6, demonstrated as the percentage of cells with the intracellular expression of cytokine (%) (independent groups were compared using Kruskal–Wallis and *post-hoc* Dunn’s multiple comparison tests. Two related groups were compared using the Wilcoxon signed-rank test). Solid horizontal lines denote statistically significant differences between similarly treated cells of different age groups, that is, older adults aged under 85 years vs. older adults aged over 85 years. Dashed horizontal lines above paired bars denote statistically significant differences between non-stimulated vs. stimulated cells within the same age group. The respective symbols denote *p <0.05 and **p <0.01.

The gating strategy used for the flow cytometric analysis of cultured PBMCs is shown in **Supplementary Figure 3**. FACS histogram overlay and consistent dot plots for IL-6 expression relative to the isotype control in unstimulated NK cells or those stimulated with IL-2, LPS, or PMA with ionomycin in representative samples of young adults, older adults aged under 85 years, and older adults aged over 85 years are presented in **Figures 3**–**5**. These results correspond to the IL-6 expression analysis presented in **Figure 6**. The expression of IL-6 in cultured, unstimulated CD3^−^CD56^+^ cells was over two times higher in the group of older adults over 85 years (36.94 ± 8.59%) when compared to the group of young adults (16.75 ± 2.63%) and older adults under 85 years (15.61 ± 3.71%). This group of adults also showed nearly three and over two times higher percentages of NK cells expressing IL-6 after stimulation with IL-2 (47.46 ± 9.25%) or PMA with ionomycin (55.3 ± 9.01%), respectively, when compared to those of the older adults under 85 years that were treated similarly with IL-2 (17.3 ± 4.9%) or PMA with ionomycin (25.63 ± 5.24%), with the differences being statistically significant (p <0.05) (**Figure 6**).

Interestingly, NK cells of older adults over 85 years that were stimulated with IL-2 (47.46 ± 9.25%) also showed a significantly higher expression of IL-6 compared to that of the control, non-stimulated cells (36.94 ± 8.59%) (p <0.01), in contrast to the NK cells of young adults and older adults under 85 years that presented only slightly increased percentages of cells expressing IL-6 after stimulation with IL-2. When NK cells from all age groups were stimulated with LPS, no significant difference in IL-6 expression was observed. However, CD3^−^CD56^+^ cells after stimulation with PMA and ionomycin showed a significant increase in the percentages of IL-6-expressing cells observed in all studied groups (p <0.01) (**Figure 6**). Scatter plots of the data are shown in **Supplementary Figure 4**.

Notably, the baseline expression levels of IL-6 in uncultured NK cells analyzed immediately after blood sample collection from young adults, older adults aged under 85 years, and older adults aged over 85 years were comparable to those of unstimulated, cultured NK cells of young adults and older adults aged under 85 years (**Supplementary Figure 5**).

### 3.4 HO-1 concentration in NK cell extracts

**Figure 7:**
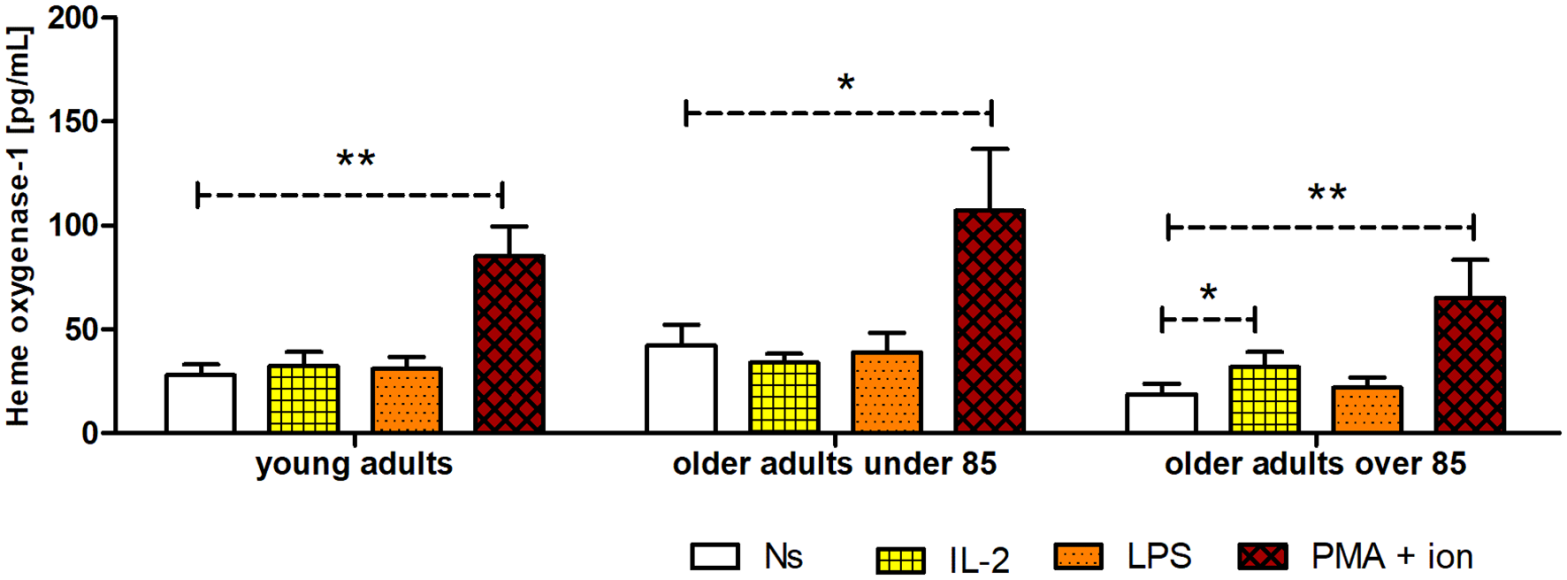
Concentration of HO-1 in cultured, non-stimulated, and stimulated (IL-2, LPS, or PMA with ionomycin) NK cells of young adults, older adults aged under 85 years, and older adults aged over 85 years. Data are presented as the mean ± SEM (independent groups were compared using the Kruskal–Wallis test, and two related groups compared using the Wilcoxon signed-rank test). Dashed horizontal lines above paired bars denote statistically significant differences between non-stimulated vs. stimulated cells within the same age group. The respective symbols denote *p <0.05 and **p <0.01.

The concentration of HO-1 was the lowest in unstimulated NK cells of the oldest group of adults (18.66 ± 5.2 pg/mL). It was 1.5 times higher in young adults (27.9 ± 5.01 pg/mL) and over two times higher in older adults under 85 years (42.2 ± 10.03 pg/mL); however, these differences were not statistically significant (**Figure 7**). Interestingly, NK cells stimulated with IL-2 (31.77 ± 7.22 pg/mL) revealed a significantly higher increase in HO-1 content compared to that of the control, unstimulated cells (18.66 ± 5.2 pg/mL) only in the group of the adults over 85 years of age (p <0.05). The stimulation of NK cells with LPS did not result in significant changes in HO-1 concentration in the studied age groups. However, NK cells showed the highest sensitivity to stimulation with PMA and ionomycin in all age groups, exhibiting a significant 2.5–3.5-fold increase in HO-1 concentration in the analyzed cell extracts. Intriguingly, the highest relative increase was observed in the group of oldest adults, as the HO-1 concentration in PMA-stimulated cells was 3.5 times higher (65.26 ± 18.15 pg/mL) compared to that of the control, non-stimulated cells (18.66 ± 5.2 pg/mL) (p <0.01) (**Figure 7**). Scatter plots of the data are shown in **Supplementary Figure 6**.

### 3.5 Relationships between parameters analyzed in NK cells and serum

Correlation analysis was performed for parameters studied in NK cells cultured for 48 h with or without stimulation with IL-2, LPS, or PMA with ionomycin (HO-1 and IL-6) and the parameters analyzed in serum (NLRP3, GSH, GSSG, and CRP). Data regarding serum CRP concentrations in the study population are presented in **Supplementary Figure 7**. Serum levels of NLRP3, GSH, GSSG, and CRP did not correlate with intracellular HO-1 concentrations. In contrast, the analysis performed on NK cells unstimulated or stimulated with the studied agents revealed a series of statistically significant weak-to-moderate negative correlations between the measured intracellular expression of IL-6 and HO-1 concentration under all tested experimental conditions (**Table 1**). Correlation scatter plots of the data are shown in **Supplementary Figure 8**.

**Table 1:** Correlation analysis of the concentration of HO-1 in cell extracts of cultured, non-stimulated, or stimulated (IL-2, LPS, or PMA with ionomycin) NK cells and the indicated parameters evaluated in the studied population.

Parameter	Stimulation type	NLRP3	GSSG	GSH	CRP	IL-6
**HO-1**	**none**	ns	ns	ns	ns	−0.35*
	**IL-2**	ns	ns	ns	ns	−0.363*
	**LPS**	ns	ns	ns	ns	−0.372*
	**PMA + ion**	ns	ns	ns	ns	−0.583***

All values are presented as statistically significant Spearman’s correlation coefficients. The respective symbols denote *p <0.05 and ***p <0.001.

CRP, C reactive protein; GSH, glutathione; GSSG, glutathione disulfide; HO-1, heme oxygenase-1; LPS, lipopolysaccharide; NLRP3, NOD-like receptor protein 3; ns, not significant; PMA, phorbol 12-myristate 13-acetate; ion, ionomycin.

## 4 Discussion

Most immune cell populations undergo changes with age that are related to their number and/or cellular activities. Aging is characterized by progressive changes in redox status due to the decrease in efficiency of endogenous antioxidant systems that occurs with age, which affects most cellular signaling pathways and impairs cellular homeostasis. The accompanied immune system dysregulation can subsequently result in chronic systemic inflammation (12). These changes may not always be harmful and can be adaptive, contributing to remodeling of the immune system to adjust to challenging environments (36). The present study contributes to a better understanding of the process of how the immune system adapts to environmental conditions associated with aging, particularly NK cells, which are important components of innate immunity.

The NLRP3 concentration in serum may be used as an additional biomarker of inflammation, which has already been applied to some inflammatory conditions, including periodontitis (37), sepsis (38), and peripheral arterial disease (39). The age groups compared in our study did not differ significantly in serum NLRP3 protein concentration, which were within the range of 3.75–4.12 ng/mL. In healthy adults, similar concentrations were observed across different studies. Yang et al. (38) and Bartoli-Leonard et al. (39) reported slightly lower concentrations ranging from 1–3 ng/mL and 0–5 ng/mL in individuals with an average age of 61.2 ± 9.6 and 59.4 ± 2.8 years, respectively. Conversely, Isola et al. (37) observed slightly higher concentrations (averaging 7.2 ± 3.6 ng/mL) in a group with an average age of 51.0 ± 2.1 years. These disparities might result from the various levels of sensitivity of the applied assays and the different ages of the volunteers involved. Interestingly, a minor increase in both groups of older adults was observed in our study when the serum NLRP3 protein concentration was measured in ng/mg total serum protein. This increase was not observed in the group of older adults over 85 years of age when the NLRP3 concentration was evaluated in ng/mL. This discrepancy might result from the fact that the total protein content in serum decreases with age (40); thus, the concentration calculated in ng/mg protein better illustrates the aging process.

GSH forms part of an enzymatic antioxidant system involved in numerous physiological processes, including aging (41). Most GSH studies have reported a significant decrease in its plasma concentration with increasing age (42, 43). Similarly, in our study, we observed a decreasing tendency in the GSH concentration that accompanied the aging process. As aging progresses, the GSSG/GSH ratio gradually shifts towards a more pro-oxidizing state, which is primarily attributed to the increased concentration of GSSG (44, 45). Correspondingly, we also observed a minor increase in the GSSG concentration with age and a higher GSSG/GSH ratio, although these changes were not statistically significant. The comparison of GSH and GSSG serum concentration values between different studies was often difficult because various authors applied different assays (42, 44), or the studies did not consider the process of aging when the same assay was applied (46). In our earlier studies on cultured NK cells isolated from PBMCs, we observed typical age-related changes in oxidative stress indices. NK cells were cultured for 48 h in the absence (control) or presence of stimulatory agents (IL-2, LPS, or PMA with ionomycin). We then measured the content of protein carbonyl groups and the concentration of 8-isoprostanes in the NK cell extracts. The highest concentrations of carbonyl groups and 8-isoprostanes were observed in young adults, significantly exceeding those in older adults. Interestingly, despite the stimulation of NK cells, we observed no significant changes in the levels of oxidative stress in all age groups studied (31).

The low-grade, chronic systemic pro-inflammatory state associated with aging results from elevated levels of pro-inflammatory cytokines, including IL-6 (47). The intracellular expression of this cytokine is usually not analyzed in NK cells as it is secreted primarily by monocytes and macrophages, and only to some degree by other cell types, including T and NK cells (24, 48). Fauriat et al. reported that low-grade IL-6 expression in resting NK cells increased after interaction with K562 cells (49). Our study additionally focused on the process of aging and showed IL-6 basal expression both in non-cultured NK cells, which were analyzed immediately after blood sample collection, and in non-stimulated, cultured NK cells. This was observed in NK cells of all studied age groups, with a more prominent increase observed in both stimulated with IL-2, LPS, or PMA with ionomycin cells and the control, unstimulated cells of adults aged over 85 years. Similarly, increased expression of IL-6 in participants aged over 85 years was found in NKT-like cells and, to a lesser extent, in T cells. Similar to NK cells, these cells also revealed IL-6 basal expression in young adults and older adults under 85 years of age (32). Interestingly, NK cells of adults over 85 years of age analyzed in the study appeared to show the highest sensitivity to stimulation, as they revealed a significant increase in IL-6 expression after stimulation with IL-2 or PMA with ionomycin and a minor increase after stimulation with LPS, in contrast to the other age groups that responded significantly only to stimulation with PMA and ionomycin. Stimulating agents were selected for this study because they activate various lymphocyte signaling pathways. IL-2 is a major growth factor for T and NK cells and is involved in the priming and activation of NK cells (50). LPS is a component of the outer membrane of gram-negative bacteria and is associated with the activation cells of both innate and adaptive immunity. It is a ligand for TLR4 receptors expressed on the surface of antigen-presenting cells and lymphocytes, including T and NK cells (51, 52). PMA is a protein kinase C activator used for the robust and non-specific stimulation of both T and NK lymphocytes in combination with ionomycin, a calcium ion channel-opening antibiotic that imitates the activity of IP3 and elevates Ca^2+^ concentrations in the cytoplasm (53).

Similar stimulation conditions were applied in the experiments to estimate the HO-1 content in NK cell extracts. To the best of our knowledge, the expression of HO-1 has not previously been analyzed in NK cells. However, some studies were performed on PBMCs. Done and coauthors analyzed both the mRNA and protein expression of HO-1 in PBMCs of older (age 63 ± 1 years) and young (age 23 ± 1 years) men within 24 h after a single session of submaximal aerobic exercise. They found that exercise elicited a significant increase in mRNA expression, but only in young adults. However, they did not observe differences between young and older adults regarding HO-1 protein expression either at the basal level or in response to exercise (54). Our study analyzed the HO-1 content in NK cell extracts after 48 h of cell culture in the presence or absence of stimulatory agents. Similarly, we did not observe significant differences in HO-1 concentrations in unstimulated NK cells among the studied age groups. However, in our study, the NK cells of adults over 85 years of age revealed the highest sensitivity to stimulation compared to that in other age groups, although they presented the relatively lowest basal expression of HO-1 (HSP32). Interestingly, our group observed a similar phenomenon for other cellular protective proteins analyzed in stimulated and non-stimulated NK cells, such as SIRT1, HSP70, and SOD2 (31). Interestingly, HSP70, HSP32, and SIRT1 were described earlier by Calabrese et al. as vitagenes, and SOD2 expression found to be regulated by SIRT1 (55). Vitagene products are involved in the maintenance of cellular homeostasis under stressful conditions, and HO-1 appears to play a crucial role in the Nrf2-dependent vitagene pathway associated with antioxidant and anti-inflammatory responses (56).

We also observed weak-to-moderate negative correlations between intracellular IL-6 expression and HO-1 concentration. These results confirmed the relationships observed earlier for hepatoma cells (22), microglial cells (57), and macrophages (58), which revealed the presence of a negative feedback loop between intracellular levels of HO-1 and the expression of IL-6. However, these relationships might differ in various cell and tissue types, as a study performed on multiple myeloma cells showed positive crosstalk between IL-6 and HO-1, in which HO-1 induced IL-6 expression (27). It was also found that in spite of the beneficial mechanisms of cytoprotection provided by HO-1 in various stress conditions, its induction might be related to the development of some undesirable processes, including carcinogenesis, metastasis, and neurodystrophic disorders (23). As the protective role of HO-1 results from an increase in survival and suppression of apoptotic pathways, it appears to play a dual role in cancer progression (59). Thus, our study contributes new data to this field of research.

The limitations of the study concern the number of participants involved and the unequal sex distribution in the age groups. We did not aim to analyze sex alterations in the samples, similar to our earlier studies (31, 32). However, sex-based changes cannot be ruled out, as some differences were found in HO-1 expression in the cardiac and aortic tissues of female and male rats (60). Another limitation is that we focused on the analysis of only one pro-inflammatory cytokine (IL-6) to check for the presence of the previously described relationships (22). However, we believe that there are also strong aspects to our study, as we applied strict exclusion criteria for study participants to focus on the healthy aging process. Thus, the analyzed parameters describing the serum oxidative/inflammatory status used in this study (NLRP3, GSH, and GSSG concentrations or the GSSG/GSH ratio) displayed values typical of the course of aging. To our knowledge, the current study is the first to concentrate on the expression of HO-1 in NK cells in general, and the first to focus on alterations observed in these stimulated and non-stimulated cells during the aging process in the context of the adaptive stress response. This study also contributes to existing research on the IL-6-secreting capacity of NK cells, as reports on this issue are scarce.

In conclusion, our study showed that NK cells of adults over 85 years of age, despite having the lowest basal levels of HO-1, had the highest sensitivity to stimulation compared to that in adults of the other age groups. The observed negative correlation between intracellular HO-1 concentration and IL-6 expression confirmed the presence of a negative feedback loop between these parameters. This phenomenon might result from the involvement of HO-1 in adaptive stress response signaling pathways and the maintenance of cellular homeostasis under stressful conditions. Moreover, this study adds to the existing knowledge on the activity of HO-1 in NK cells, a crucial component of innate immunity, during the process of aging and shows, for the first time, how its levels change after activation in different age groups. The IL-6 secretory pattern of NK cells under various stimulatory conditions also seems to be of significant value and adds to the novelty of this study, especially in the context of HO-1 and IL-6 relationships.

## Data availability statement

The raw data supporting the conclusions of this article will be made available by the authors, without undue reservation.

## Ethics statement

The studies involving humans were approved by Bioethics Committee for Scientific Research of the Medical University of Gdańsk, Poland. The studies were conducted in accordance with the local legislation and institutional requirements. The participants provided their written informed consent to participate in this study.

## Author contributions

LK: Conceptualization, Data curation, Funding acquisition, Investigation, Methodology, Visualization, Writing – original draft, Writing – review & editing. JK: Conceptualization, Investigation, Methodology, Writing – review & editing. MK: Investigation, Methodology, Writing – review & editing. JF: Investigation, Resources, Writing – review & editing. ZK: Funding acquisition, Supervision, Writing – review & editing.
