# Supplementary material for: Expression of a stress-inducible heme oxygenase-1 in NK cells is maintained in the process of human aging
Source: Front Immunol. 2024 Jul 19;15:1398468. doi: 10.3389/fimmu.2024.1398468 (PMC11294084; doi:10.3389/fimmu.2024.1398468)
Supplement: Supplementary file 1 [file DataSheet_1.docx]

Supplementary Material

# Supplementary Figures


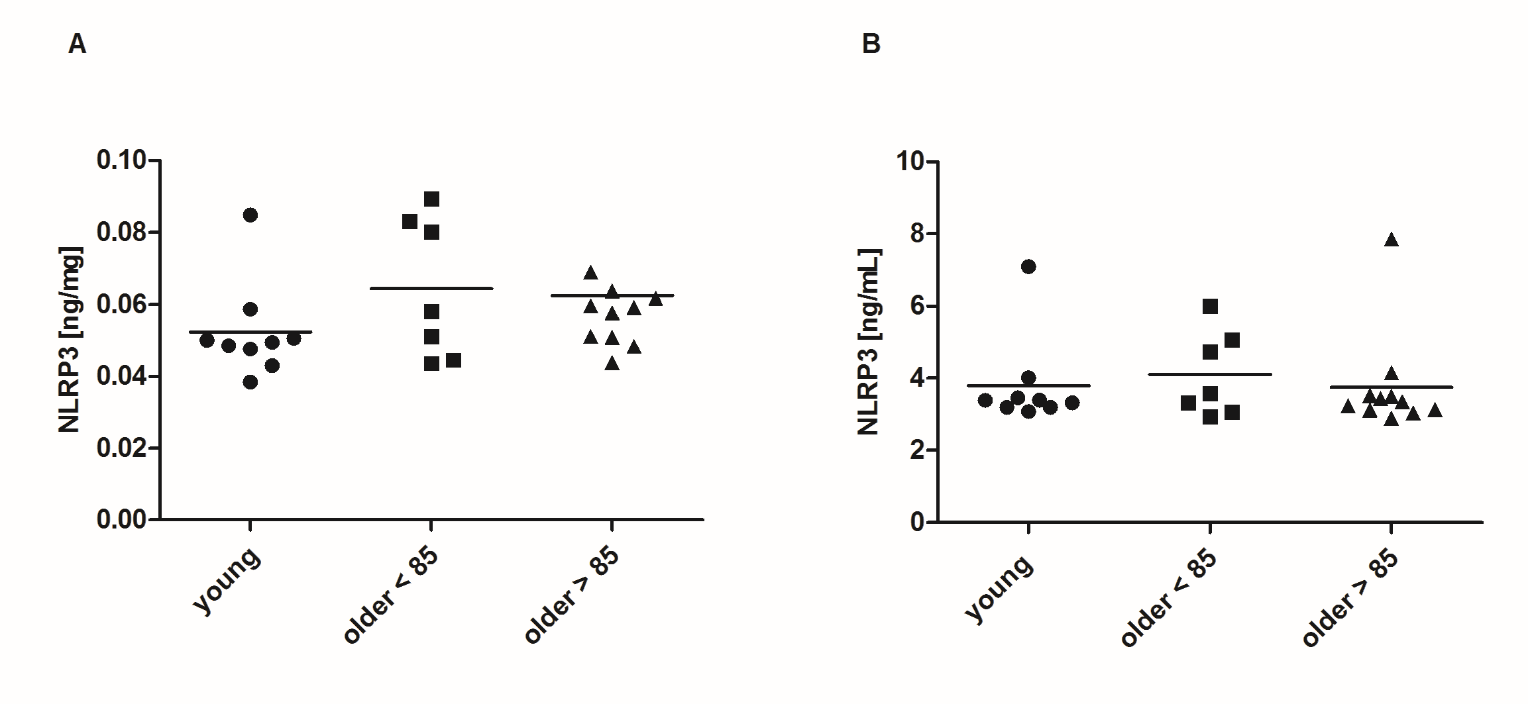


**Supplementary Figure 1.** NLRP3 concentrations measured in sera of young adults (dots), older adults aged under 85 years (squares) and older adults aged over 85 years (triangles). The horizontal line indicates the mean; p>0.05 (data compared using the Kruskal-Wallis test). (**A)** Concentration of NLRP3 in serum presented in ng/mg protein. (**B)** Serum NLRP3 concentration presented in ng/mL.





**Supplementary Figure 2.** Selected parameters of the antioxidant/oxidant system determined in sera of young adults (dots), older adults aged under 85 years (squares) and older adults aged over 85 years (triangles). The horizontal line indicates the mean; p>0.05 (data compared using the Kruskal-Wallis test). **(A)** Concentration of glutathione (GSH) in serum (µM). (**B)** Concentration of glutathione disulfide (GSSG) in serum (µM). (**C)** Serum GSSG/GSH ratio.

^
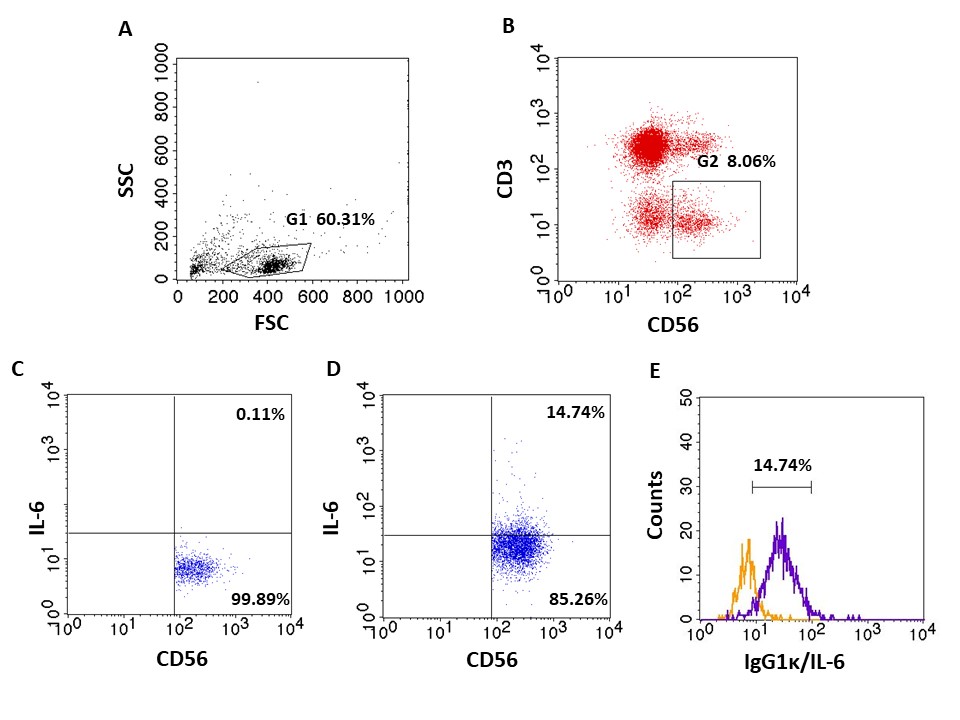
^

**Supplementary Figure 3** . Gating strategy performed for flow cytometric analysis of NK cells (CD3^-^CD56^+^). **(A)** Lymphocyte gating in the population of PBMCs, lymphocytes were defined as FSC_low_/SSC_low_ cells (G1). **(B)** NK cell gating, NK cells were defined as the CD3 negative (CD3^-^) and CD56 positive (CD56^+^) cells (G2). **(C)** Isotype control for IL-6 positive NK cells. **(D)** NK cells expressing IL-6 were identified in the upper right quadrant. **(E)** IL-6 expression (purple line) vs isotype control (orange line) presented in NK cells. All the studied age groups were analyzed with the same gating strategy.


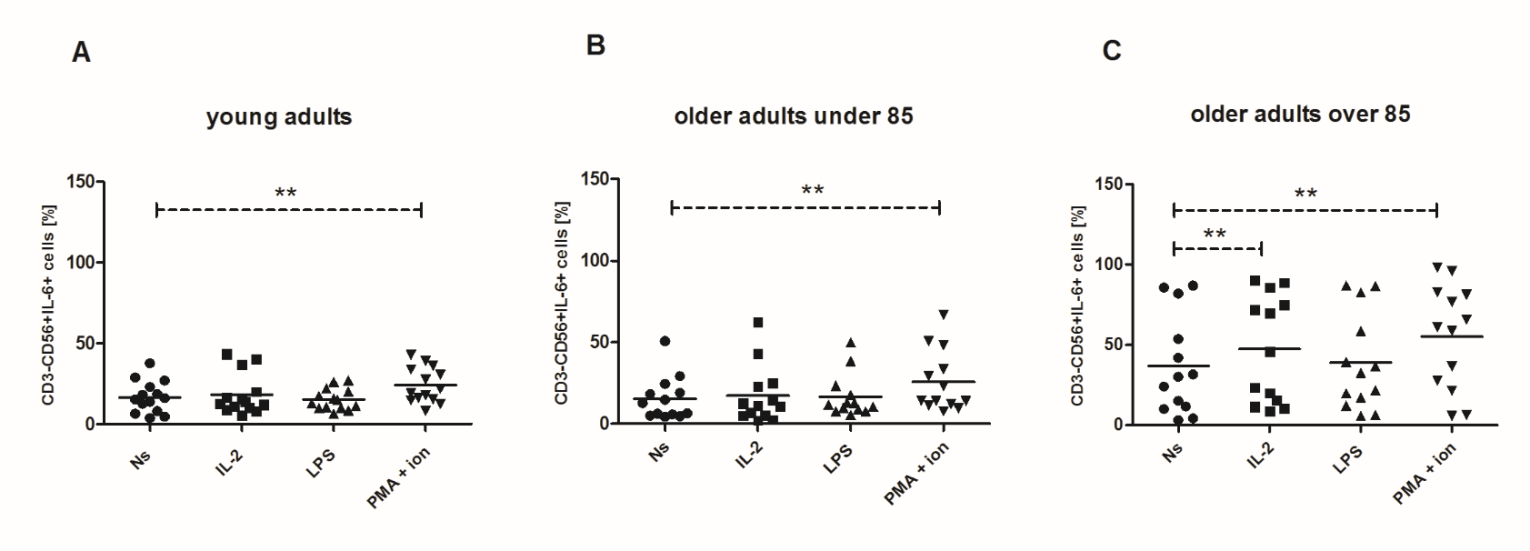


**Supplementary Figure 4.** Expression of IL-6 in cultured, non-stimulated (dots) and stimulated with IL-2 (squares), LPS (triangles), or PMA with ionomycin (reversed triangles) NK cells of young adults (**A**), older adults aged under 85 years (**B**) and older adults aged over 85 years (**C**). The horizontal solid line indicates the mean. The expression of IL-6 is demonstrated as the percentage of cells with the intracellular expression of cytokine (%). Two related groups were compared using the Wilcoxon signed rank test. Dashed horizontal lines indicate statistically significant differences between unstimulated vs. stimulated cells within the same age group. The asterisks denote ** p < 0.01.


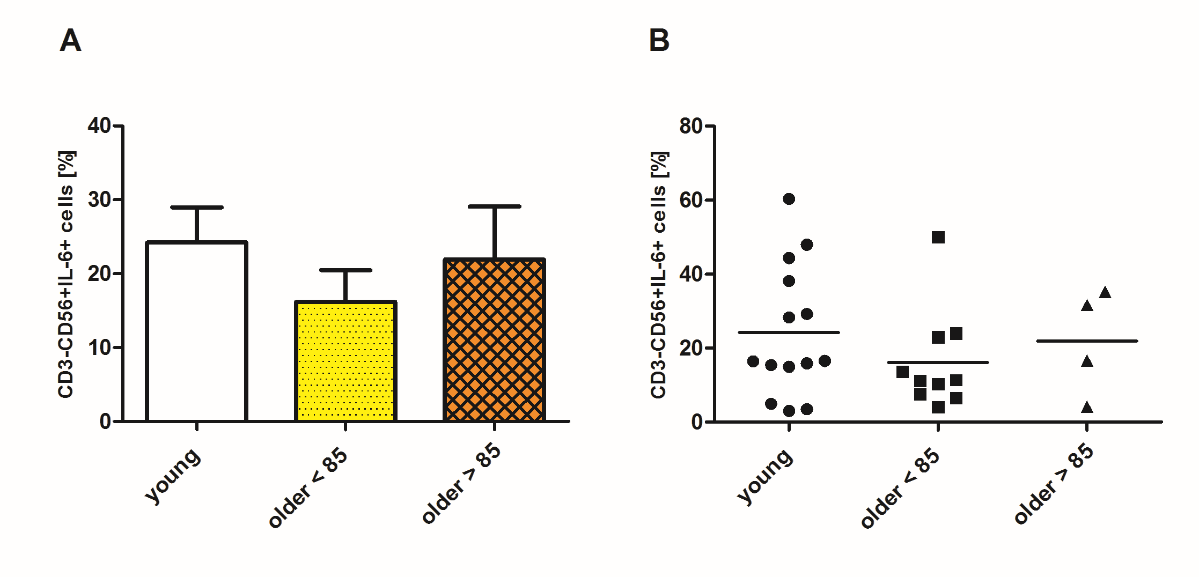


**Supplementary Figure 5.** Expression of IL-6 in uncultured, analyzed directly after blood sample collection NK cells of young adults, older adults aged under 85 years and older adults aged over 85 years. The expression of IL-6 is demonstrated as the percentage of cells with the intracellular expression of cytokine (%). (**A**) Data are presented as the mean ± SEM (**B**) Data are presented as scatter plots with a horizontal solid line indicating the mean; p>0.05 (groups were compared using the Kruskal-Wallis test).





**Supplementary Figure 6.** Concentration of heme oxygenase 1 (HO-1) in cultured, non-stimulated (dots) and stimulated with IL-2 (squares), LPS (triangles), or PMA with ionomycin (reversed triangles) NK cells of young adults (**A**), older adults aged under 85 years (**B**) and older adults aged over 85years (**C**). The horizontal solid line indicates the mean. Two related groups were compared using the Wilcoxon signed rank test. Dashed horizontal lines indicate statistically significant differences between non-stimulated vs. stimulated cells within the same studied age group. The asterisks denote * p < 0.05 and ** p < 0.01.





**Supplementary Figure 7.** Concentration of C reactive protein (CRP) in serum of young adults, older adults aged under 85 years and older adults aged over 85 years. (**A**) Data are presented as the mean ± SEM. The solid horizontal line denotes statistically significant difference between the studied age groups. (**B**) Data are presented as scatter plots with a horizontal solid line indicating the mean. Dashed horizontal line shows statistically significant difference between the studied age groups (data were analyzed using the Kruskal-Wallis test and posthoc Dunn’s multiple comparison test). The asterisk denotes * p < 0.05





**Supplementary Figure 8.** Correlation scatter plots showing relationships between the concentration of HO-1 in cell extracts of cultured, non-stimulated (Ns) (**A**), stimulated with IL-2 (**B**), LPS (**C**), or PMA with ionomycin (PMA + ion) (**D**) NK cells and the expression of IL-6 in respectively treated NK cells demonstrated as the percentage of CD3^-^CD56^+^ cells with the intracellular expression of the analyzed cytokine in the studied population. All data are presented as statistically significant Spearman’s correlation coefficients (Rs) with corresponding p values.

**
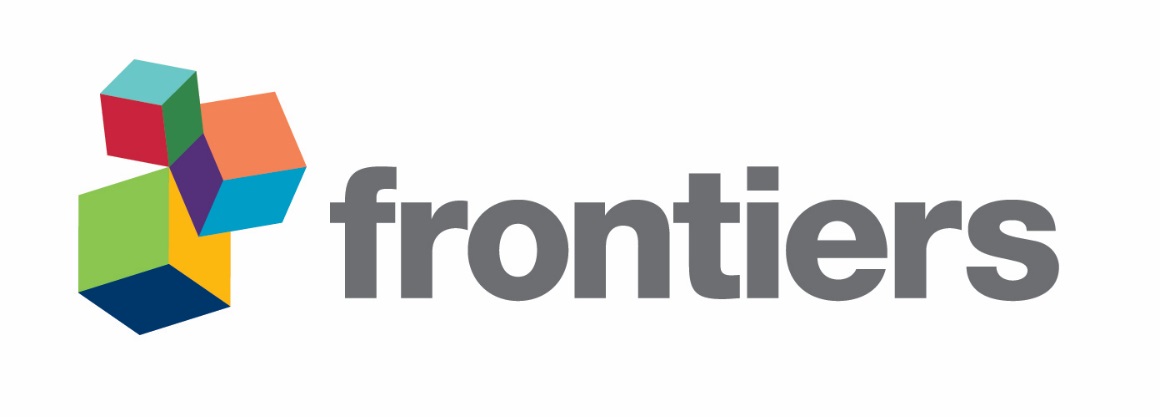
**
